# Ileostomy closure by colorectal surgeons results in less major morbidity: results from an institutional change in practice and awareness

**DOI:** 10.1007/s00384-015-2478-1

**Published:** 2016-01-05

**Authors:** G. D. Musters, J. J. Atema, H. L. van Westreenen, C. J. Buskens, W. A. Bemelman, P. J. Tanis

**Affiliations:** Department of Surgery, Academic Medical Centre, University of Amsterdam, Post Box 22660, 1105 AZ Amsterdam, The Netherlands; Department of Surgery, Isala Clinics, Zwolle, The Netherlands

**Keywords:** Ileostomy reversal, Ileostomy closure, Ileostomy, Complications, Morbidity

## Abstract

**Purpose:**

Previous institutional analysis of ileostomy closure revealed substantial morbidity. This subsequent study aimed at determining if a change in clinical practice resulted in reduced complication rates.

**Methods:**

Between June 2004 and January 2014, all consecutive adult patients undergoing ileostomy closure were retrospectively identified. Postoperative outcome after change in clinical practice consisting of routine participation of a colorectal surgeon, stapled side-to-side anastomosis and increased clinical awareness (cohort B) was compared with our previously published historical control group (cohort A). The primary outcome was major morbidity, defined as Clavien-Dindo grade three or higher. Independent risk factors of major morbidity were identified using multivariable analysis.

**Results:**

In total, 165 patients underwent ileostomy closure in cohort A, and 144 patients in cohort B. At baseline, more primary diverting ileostomies were present in cohort A (94 vs. 82 %; *p* = 0.001) with a similar rate of loop and end-ileostomy between the two cohorts (*p* = 0.331). A significant increase in colorectal surgeon participation (89 vs. 53 %; *p* < 0.001) and stapled side-to-side anastomosis was observed (63 vs. 16 %; *p* < 0.001). The major morbidity rate was 11 % in cohort A, which significantly reduced to 4 % in cohort B (*p* = 0.03). Surgery being performed or supervised by a colorectal surgeon (odds ratio [OR] 0.28, 95 % CI 0.11–0.67) and loop-ileostomy compared to end-ileostomy (OR 0.18, 95 % CI 0.07–0.52) were independently associated with lower major morbidity.

**Conclusion:**

Ileostomy closure appears to be more complex surgery then generally considered, especially end-ileostomy closure. Postoperative outcome could be significantly improved by a change in surgical practice.

**Electronic supplementary material:**

The online version of this article (doi:10.1007/s00384-015-2478-1) contains supplementary material, which is available to authorized users.

## Introduction

Defunctioning ileostomies are constructed for several reasons, but mostly to protect a low colorectal, colo-anal or an ileal pouch-anal anastomosis (IPAA). Although there is still debate about the value of routine diversion of such anastomoses, a defunctioning ileostomy has been reported to significantly reduce clinical anastomotic leakage rates and mitigate its consequences [1]. In patients who develop leakage of a non-diverted anastomosis, a secondary defunctioning ileostomy can be constructed if breakdown of the anastomosis is not indicated. Less frequent indications for diverting ileostomy are inflammatory bowel disease, intestinal ischemia, oncological diseases, functional problems or surgical complications other than distal leaking anastomosis.

Although a defunctioning ileostomy has clear advantages, stoma-related morbidity should be taken into account as well. Closure of a defunctioning ileostomy can result in substantial morbidity, with a reported complication rate of 17 % in a systematic review of more than 6000 patients [2]. A retrospective cohort study of ileostomy closures performed at our own university hospital revealed a morbidity rate of 20 %, with postoperative small bowel obstruction as the most frequent complication [3]. As a result, increasing the surgical expertise present at closure and improving the perioperative clinical awareness of potential risks changed our clinical practice.

The aim of this study was to evaluate if these changes in practice and increased awareness of the associated risks of the procedure led to a reduced major morbidity after ileostomy closure. Secondly, we aimed to identify independent risk factors of major morbidity following ileostomy closure.

## Methods

### Patients and management

All consecutive adult patients undergoing ileostomy closure between June 2004 and January 2014 were identified from a surgical administrative database. Patients were included in the present analysis independent of the underlying disease or the indication for constructing an ileostomy. Patients who underwent ileostomy closure between June 2004 and June 2010 (cohort A) were compared with patients operated upon between July 2010 and January 2014 (cohort B). Cohort A comprises of patients who were also described in a previous study [3].

Antibiotic prophylaxis was routinely given pre-operatively. Bowel continuity was restored by either a hand-sewn anastomosis using a running PDS 3.0 suture in an end-to-end, side-to-end, or end-to-side configuration, or by a side-to-side stapled anastomosis. The fascias of the posterior and anterior rectus sheath were separately closed by running or interrupted sutures using Vicryl or PDS. The skin was partially closed with approximating interrupted transcutaneous sutures or with a purse string intracutaneous suture. Oral intake was started at day one postoperatively if tolerated. No routine imaging was performed to evaluate incisional hernia at the previous stoma site.

Evaluation of our results in 2010 revealed considerable morbidity after ileostomy closure and resulted in several management changes [3]. Firstly, the operating team was more strictly selected based on specific expertise. Before June 2010, a surgical resident predominantly performed ileostomy closure and was not being routinely supervised by a consultant. When a resident was supervised, the supervising surgeon could be any type of surgical consultant. After June 2010, ileostomy closure was preferably performed or supervised by a colorectal surgeon, being either a consultant or fellow. There is not an official colorectal subspecialisation within gastrointestinal surgery in the Netherlands, but these consultants and fellows almost exclusively perform colorectal surgery in daily practice at the AMC. Secondly, side-to-side stapled anastomoses were constructed whenever possible, instead of hand-sewn end-to-end anastomoses. Besides these changes in surgical practice, the overall awareness of the risk of major morbidity after ileostomy reversal increased.

Since the present study involved a retrospective analysis of data, Dutch regulations do not require written informed consent.

### Data collection

Patient and treatment characteristics were retrospectively collected from patient records.

Operative reports, radiology reports and patient charts were used for collection of data on patient’s demographics, primary treatment characteristics, operative technique of stoma closure, 30-day postoperative complications, hospital stay and out-patient follow-up with respect to long-term stoma site-related complications.

### Definition of outcome

The primary endpoint was major morbidity within 30 days postoperatively, defined as all complications classified as Clavien-Dindo grade three or higher. This includes a complication for which a surgical, endoscopic or radiological intervention is required (grade three), a life threatening complication for which intensive care management is needed (grade four) or when the patient dies (grade five) [4].

### Statistical analysis

According to distribution, descriptive data were reported as median with interquartile range (IQR) or mean ± standard deviation (SD). Categorical data were analysed with the Chi-square-test or Fisher’s exact test, and continuous variables were analysed using the Mann-Whitney *U* test or the independent *T* test, according to the distribution. Univariable logistic regression analysis was performed to identify risk factors for major morbidity following ileostomy closure. All predictors with a *P* value of less than 0.10 in univariable analysis were candidate variables for inclusion in a multivariable model. Multivariable regression analysis was used to identify independent risk factors for major morbidity after stoma reversal. All analyses were performed with IBM SPSS statistics, version 20.0.0 (IBM Corp., Armonk, NY, United States).

## Results

### Patients and stoma characteristics

Between June 2004 and January 2014, 309 patients underwent ileostomy closure and were included in the present study. Cohort A consisted of 165 patients, and cohort B consisted of the remaining 144 patients who were operated after July 2010.

Patient and stoma characteristics of both the cohorts are displayed in Table 1. The primary diagnoses differed significantly between the two cohorts; ulcerative colitis was more frequently diagnosed in the cohort A (35.8 vs 16.7 %), while more patients had a stoma for other indications in the cohort B (15.2 vs 26.4 %; *p* = 0.003). Furthermore, patients in the cohort A were more often classified as grade III according to the American Society of Anaesthesiologists (ASA) (18.2 vs 7.6 %; *p* = 0.024). Primary surgery was more often performed using an open approach (71.5 vs 46.5 %; *p* < 0.001) and more often with diversion during the initial surgery (93.9 vs 81.9 %; *p* = 0.001) in the cohort A, compared with the cohort B.

**Table 1:** Patients and stoma characteristics

		Cohort A	Cohort B	*p* value
		June 2004–June 2010	July 2010–Jan 2014	
		(*n* = 165)	(*n* = 144)	
Gender	Males (*n*, %)	93 (56.4)	83 (57.6)	0.821
Age	Mean age (years, ±SD)	48.9 (±16.5)	51.4 (±15.0)	0.474
BMI	Mean BMI (kg/m^2^ ± SD)	24.9 (±5.9)	24.3 (±5.0)	0.411
Smoking				<0.001
	Number of patients (%)	30 (18.2)	21 (14.6)	
	Unknown (*n*, %)	52 (31.5)	10 (7.0)	
ASA classification				0.024
	ASA I (*n*, %)	32 (19.4)	32 (22.2)	
	ASA II (*n*, %)	103 (62.4)	101 (70.1)	
	ASA III (*n*, %)	30 (18.2)	11 (7.6)	
Primary diagnosis				0.003
	Colorectal cancer (*n*, %)	61 (36.9)	60 (41.7)	
	Ulcerative colitis (*n*, %)	59 (35.8)	24 (16.7)	
	Morbus Crohn (*n*, %)	9 (5.5)	9 (6.3)	
	Familial adenomatous polyposis (*n*, %)	11 (6.7)	13 (9.0)	
	Other (*n*, %)	25 (15.2)	38 (26.4)	
Primary surgery				<0.001
	Laparoscopic surgery (*n*, %)	47 (28.5)	77 (53.5)	
	Open surgery (*n*, %)	77 (46.7)	66 (45.8)	
	Unknown (*n*, %)	3 (1.8)	1 (0.7)	
	Low anterior resection (*n*, %)	52 (31.5)	45 (31.3)	0.004
	IPAA (*n*, %)	79 (47.9)	47 (32.6)	
	Sigmoid resection (*n*, %)	12 (7.2)	14 (9.7)	
	Diversion without resection (*n*, %)	11 (6.6)	14 (9.7)	
	Colonic resection (*n*, %)	10 (6.0)	16 (11.1)	
	Small bowel resection (*n*, %)	1 (0.6)	1 (0.7)	
	Pull through with colo-anal anastomosis (*n*, %)	0	7 (4.8)	
Indication for ileostomy				0.001
	Diversion for primary disease or during primary surgery (*n*, %)	155 (93.9)	118 (81.9)	
	Secondary diversion for anastomotic leakage (*n*, %)	10 (6.1)	26 (18.1)	
Type of ileostomy				0.331
	Loop-ileostomy (*n*, %)	152 (92.1)	128 (88.9)	
	End-ileostomy (*n*, %)	13 (7.9)	16 (11.1)	

*BMI* body mass index, *ASA* American Society of Anaesthesiology, *IPAA* ileal pouch-anal anastomosis

### Ileostomy closure characteristics

Time to ileostomy closure was comparable between both the cohorts; 22.8 (SD ±18.45) weeks in the cohort A, versus 21.6 (SD ±14.4) weeks in the cohort B (*p* = 0.09; Table 2). Stoma closure was performed or supervised by a colorectal surgeon in 53.3 % (88/165) in the cohort A, which was significantly lower compared to 88.9 % (128/144) of the patients in the cohort B (*p* < 0.001). Also, stapled anastomoses were more often constructed in cohort B (66.0 vs 10.9 %; *p* < 0.001). Stoma site closure was different from the protocol in nine patients, four patients in the cohort A and five patients in the cohort B: local gentamicin was used in five patients, and an absorbable mesh was used for fascial closure in four patients. A loop-ileostomy was closed in 92.1 % of the patients (152/165) in the cohort A and in 88.9 % of the patients (128/144) in the cohort B (*p* = 0.331).

**Table 2:** Ileostomy closure characteristics

		Cohort A	Cohort B	*p* value
		June 2004–June 2010	July 2010–Jan 2014	
		(*n* = 165)	(*n* = 144)	
Time to stoma reversal	Mean weeks (±SD)	22.8 (±18.5)	21.6 (±14.4)	0.088
Colorectal surgeon performing or supervising surgery				<0.001
	Yes (*n*, %)	88 (53.3)	133 (92.4)	
	No (*n*, %)	77 (46.7)	11 (7.6)	
Type of constructed anastomosis				<0.001
	End-to-end anastomosis (*n*, %)	117 (70.9)	40 (27.8)	
	Side-to-side anastomosis (*n*, %)	27 (16.4)	91 (63.2)	
	Side-to-end or end-to-side anastomosis (*n*, %)	8 (4.8)	6 (4.2)	
	Unknown (*n*, %)	13 (7.9)	7 (4.9)	
Anastomotic technique				<0.001
	Sewn (*n*, %)	141 (85.5)	42 (29.2)	
	Stapled (*n*, %)	18 (10.9)	95 (66.0)	
	Unknown (*n*, %)	6 (3.6)	7 (4.9)	
Skin closure				<0.001
	Purse string intracutaneous suture (*n*, %)	25 (15.2)	76 (52.8)	
	Primary closure (*n*, %)	20 (12.1)	14 (9.7)	
	Approximating interrupted transcutaneous sutures (*n*, %)	101 (61.2)	43 (29.9)	
	Unknown (*n*, %)	19 (11.5)	11 (7.6)	

### Morbidity after ileostomy closure

Thirty-day postoperative morbidity after ileostomy closure in the two cohorts is shown in Table 3. In total, 15 patients developed a wound infection (4.9 %), with a similar rate among the two cohorts. Wound infection rates were 2 % (2/101) after purse string closure, 6.3 % (9/144) using approximating interrupted transcutaneous sutures and 5.9 % (2/34) after primary closure (*p* = 0.277, missing two). Anastomotic leakage rate was 6.7 % (11/165) in the cohort A, which was significantly higher than a 2.1 % (3/144) leakage rate in the cohort B (*p* = 0.05). Major morbidity rate was also significantly higher in the cohort A (10.9 %; 18/165) compared to that in the cohort B (4.2 %; 6/144) (*p* = 0.03). Major morbidity occurred in 9.3 % (17/183) of the patients with a hand-sewn anastomosis and in 5.3 % (6/113) of the patients with a stapled anastomosis (*p* = 0.214). Similarly, anastomotic leakage (5.5 vs. 2.7 %; *p* = 0.83) and postoperative ileus (9.8 vs. 3.5 %; *p* = 0.07) showed non-significant differences between hand-sewn and stapled anastomoses, respectively.

**Table 3:** Postoperative morbidity after ileostomy closure

			Cohort A	Cohort B	*p* value
			June 2004–June 2010	July 2010–Jan 2014	
			(*n* = 165)	(*n* = 144)	
Hospital stay		Median days (±IQR)	5.0 (3–6)	5.0 (4–7)	0.86
Wound infection		Number of patients (%)	7 (4.2)	8 (5.5)	0.59
Ileus		Number of patients (%)	15 (9.1)	7 (4.8)	0.15
Abscess		Intra-abdominal (*n*, %)	4 (2.4)	3 (2.1)	0.84
Anastomotic leakage		Number of patients (%)	11 (6.7)	3 (2.1)	0.05
Major morbidity					
	Clavien-Dindo ≥3 (*n*, %)		18 (10.9)	6 (4.2)	0.03
	Clavien-Dindo grade 1–2 (*n*, %)		12 (7.2)	11 (7.6)	0.90
	Clavien-Dindo grade 3 (*n*, %)		14 (8.4)	1 (0.7)	<0.01
	Clavien-Dindo grade 4 (*n*, %)		3 (1.8)	4 (2.8)	0.48
	Clavien-Dindo grade 5 (*n*, %)		1 (0.6)	1 (0.7)	0.92
Reoperation	Number of patients (%)		11 (6.7)	3 (2.0)	0.06
Readmission	Number of patients (%)		9 (5.5)	5 (3.5)	0.40

Two patients died within 30 days due to a complicated ileostomy reversal, one patient in each group. Cause of death was bleeding from the epigastric vessels, which resulted in a low flow state with cardiac ischemia and an intra-abdominal haematoma with bowel ischemia, and one patient died due to sepsis because of anastomotic leakage.

In univariable analysis, primary laparoscopic surgery and a loop-leostomy were significantly associated with a lower risk of major morbidity, compared to primary open surgery and end-ileostomy, respectively (Table 4). Furthermore, major morbidity occurred significantly less after ileostomy closure when performed or supervised by a colorectal surgeon. In multivariable analysis, loop-ileostomy and surgery performed or supervised by a colorectal surgeon remained independent favourable factors for the risk of major morbidity.

**Table 4:** Uni- and multivariable analysis of risk factors for major morbidity after ileostomy closure

	Univariable analysis			Multivariable analysis		
	OR	95 % CI	*p* value	OR	95 % CI	*p* value
Male gender	0.35	0.30–1.53	0.348	–	–	–
Age (years)	1.01	0.98–1.04	0.337	–	–	–
ASA Classification						
I	–	–	–	–	–	–
II	0.66	0.24–1.81	0.417	–	–	–
III	1.66	0.50–5.54	0.412	–	–	–
Smoking	0.34	0.08–1.50	0.155	–	–	–
BMI > 30 kg/m^2^	0.99	0.28–3.50	0.983	–	–	–
Malignant disease (versus benign disease)	0.70	0.29–1.68	0.427	–	–	–
Primary laparoscopic surgery (versus open)	0.35	0.14–0.93	0.035	0.42	0.15–1.17	0.097
Secondary diversion for anastomotic leakage (versus primary diversion)	2.04	0.71–5.82	0.183	–	–	–
Loop-ileostomy (versus end ileostomy)	0.21	0.08–0.57	0.002	0.20	0.07–0.53	0.002
Skin closure (versus primary closure)	–	–	–	–	–	–
Purse string intracutaneous suture	0.31	0.07–1.31	0.111	–	–	–
Approximating interrupted transcutaneous sutures	0.50	0.14–1.73	0.274	–	–	–
Colorectal surgeon (versus any surgeon or resident)	0.31	0.13–0.71	0.005	0.32	0.13–0.75	0.009
Stapled anastomosis (versus hand-sewn)	0.65	0.25–1.61	0.346	–	–	–
S-S anastomosis (versus S-E, E-S, E-E anastomosis)	0.92	0.38–2.22	0.863	–	–	–

*BMI* Body Mass Index, *ASA* American Society of Anaesthesiology, *S-S* side-to-side, *S-E* side-to-end, *E-S* end-to-side, *E-E* end-to-end

### Follow-up

The median follow-up after ileostomy reversal was 71.0 (IQR 14–200) weeks. After stoma reversal, a stoma was constructed again in 6.9 % (10/144) of the patients in the cohort A and in 10.9 % (18/165) in the cohort B (*p* = 0.23). This secondary stoma was constructed due to anastomotic leakage (*n* = 9), fistula (*n* = 4), incontinence (*n* = 3), construction of a pouch (*n* = 3), progression of the primary disease (*n* = 4), ileus (*n* = 2), bowel perforation (*n* = 2) and unknown reason (*n* = 1). A clinical diagnosed stoma site hernia occurred in 6.3 % (9/144) of the patients in the cohort A and in 4.2 % (7/165) of the patients in the cohort B (*p* = 0.43). A stoma site hernia occurred after a median of 9.5 (IQR 4.7–17.5) months, and in 50.0 % (8/16) of the patients, a surgical correction was performed.

## Discussion

This analysis shows that increased awareness of the risks of morbidity after ileostomy closure and adapting surgical practice significantly improved postoperative outcome. Stoma reversal being performed or supervised by a colorectal surgeon and closure of a loop-ileostomy instead of an end-ileostomy were independently associated with a lower risk of major 30-day postoperative morbidity, with odds ratios of 0.19 and 0.32, respectively.

Increasing the role of a colorectal surgeon in performing ileostomy closure resulted in a significantly decreased major morbidity rate. This might be the result of a difference in surgical technique and surgical volume between colorectal surgeons and general surgeons, surgeons with another specialisation or unsupervised residents. This is a remarkable finding, because surgical differentiation and volume is often considered to be only relevant in high complex and/or low volume surgical procedures. However, ileostomy closure should probably be regarded as redo bowel surgery, requiring meticulous dissection and adequate tissue handling within a previously exposed operating field with scar tissue. Adequate exposure of the operative field may be challenging while operating through a small hole, especially in obese patients. Several problems may be encountered during stoma closure, such as restricted length of the mesentery, bleeding from epigastric vessels in the rectus sheath and atrophy of the efferent loop. Closure of the stoma site requires adequate recognition of the abdominal wall structures and layered closure. Furthermore, experience with larger abdominal wall defects and additional reconstruction techniques are required. Therefore, restricting ileostomy reversal to a selective group of specialised surgeons might decrease complication rates.

Literature regarding complex colorectal surgery already showed that higher surgical volume results in fewer definitive stomas, better overall survival in colorectal cancer patients and lower costs [5, 6]. Whether specialised high volume surgeons are performing the procedure themselves or have a supervising role does not seem to influence the outcome. A trainee who is supervised by a colorectal surgeon can obtain similar quality results [7]. However, literature on surgical and hospital volume is not conclusive, and the definition of high volume differs substantially between studies [8].

End-ileostomy was an independent predictor of major morbidity. A possible explanation might be that identification and mobilisation of the distal limb is more complicated because of its intra-abdominal localisation. This may require an extension of the stoma site opening, or even a midline laparotomy. Furthermore, a laparoscopic approach during index surgery seemed to be a predictor of reduced morbidity after stoma closure in univariable analysis. However, primary laparoscopic surgery did not turn out to be an independent predictor in multivariate analysis anymore. This might be the result of the increased use of laparoscopic surgery over time, without a beneficial impact itself on stoma reversal morbidity.

Postoperative ileus has been reduced from 9 to 5 % over time, which might be the result of the increased use of a stapler device with construction of a side-to-side anastomosis. Because the distal limb is not functional for some time, the anastomosis is generally made to a relative small calibre distal limb if restored in an end-to-end configuration. Perioperative oedema might therefore compromise the luminal diameter causing an early bowel obstruction. With the stapled anastomosis, a greater calibre anastomosis is made which potentially reduces the risk of postoperative small bowel obstruction [9]. Although subgroup analysis did not definitively confirm this hypothesis, a non-significant trend in favour of stapled anastomosis was observed.

Besides the intraoperative measures, we postulated that increased awareness during the whole hospital admission has likely contributed to the improved outcome. It is hard to provide objective data to support this hypothesis, but this is something that is very well known from quality improvement programs using auditing. Auditing in itself is able to improve outcome, just because of getting insight into the process and being aware of items that need special attention.

Considering the potential risks associated with closure of a defunctioning ileostomy, one should critically look at its application as a routine after colo-anal and ileo-anal anastomoses. An anastomotic leakage can significantly increase short- and long-term morbidity, reduce the quality of life and might even increase the risk of local cancer recurrence [10, 11]. Preventing this severe complication seems to be attractive, but the question is what the role is of primary diversion. Besides the risk of complications associated with the presence of a stoma (i.e., dehydration, parastomal hernia, prolaps), the reversal of the stoma is associated with an additional risk of morbidity which has been described in literature to occur in up to 17 % of the patients [12]. In addition, a primary defunctioning stoma is not reversed in a substantial number of patients after LAR and also after IPAA surgery [13, 14]. A definitive stoma might reduce the quality of life and might lead to an increase in medical costs. Therefore, a more selective approach for a defunctioning ileostomy is advocated [15]. However, more research is needed to enable more appropriate patient selection.

This study is limited due to the retrospective and non-randomised design, which might have resulted in the omission of data and selection bias. Although a randomised controlled trial would be methodologically superior to a comparative cohort study, randomisation between unselected trainees and surgeons versus colorectal surgeons performing ileostomy reversal would be unethical. Another risk of bias in this retrospective cohort study was the limited number of events for a multivariate analysis, which might have led to an underestimation of the independent variables. In addition, the primary disease of ulcerative colitis significantly differed between both the groups. However, the effect of this significant baseline difference on the primary outcome might be limited because ileostomy reversal was usually performed when the patient no longer used immunosuppressive medications. Despite these limitations, the present study clearly illustrates that ileostomy reversal is probably a more complex procedure than often considered, with a not negligible risk of major morbidity. Increasing the role of a colorectal surgeon might improve outcome after ileostomy reversal.

## Electronic supplementary material

ESM 1 (DOC 100 kb)
