# Supplementary material for: Ileostomy closure by colorectal surgeons results in less major morbidity: results from an institutional change in practice and awareness
Source: Int J Colorectal Dis. 2016 Jan 5;31:661–7. doi: 10.1007/s00384-015-2478-1 (PMC4773497; doi:10.1007/s00384-015-2478-1)
Supplement: Supplementary file 1 — (DOC 100 kb) [file 384_2015_2478_MOESM1_ESM.doc]

| **Appendix 1** Patients characteristics; loop ileostomy only | | ***Cohort A***  *June 2004- June 2010*  *(n=152)* | ***Cohort B***  *July 2010- Jan 2014*  *(n=128)* |  |
| --- | --- | --- | --- | --- |
| **Gender** | *Males (n, %)* | 88 (57.9) | 74 (57.8) | 0.989 |
| **Age** | *Mean age (years, ± SD)* | 49.1 (±16.7) | 52.1 (±15.2) | 0.119 |
| **BMI^a^** | *Mean BMI (± SD)* | 24.6 (±5.5) | 24.6 (±5.0) | 0.952 |
| **Smoking** | *Number of patients (%)*  *Unknown (n, %)* | 29 (28.2)  49 (32.2) | 19 (15.7)  7 (5.4) | 0.024 |
| **ASA-classification** | *ASA I (n, %)* | 29 (19.1) | 30 (23.4) | 0.033 |
|  | *ASA II (n, %)* | 95 (62.5) | 88 (68.8) |  |
|  | *ASA III (n, %)* | 28 (18.4) | 10 (7.8) |  |
| **Primary diagnosis** | *Colorectal Cancer (n, %)* | 56 (36.8) | 55 (43.0) | 0.002 |
|  | *Ulcerative colitis* | 58 (38.2) | 23 (18.0) |  |
|  | *Morbus Crohn (n, %)* | 6 (3.9) | 4 (3.1) |  |
|  | *Familial adenomatous polyposis (n, %)* | 11 (7.2) | 13 (10.2) |  |
|  | *Other (n, %)* | 21 (13.8) | 33 (25.8) |  |
| **Primary surgery** | *Laparoscopic surgery (n, %)* | 46 (30.7) | 70 (55.1) | <0.001 |
|  | *Open surgery (n, %)* | 104 (69.3) | 57 (44.9) |  |
|  | *Unknown (n, %)* | 2 (1.3) | 1 (0.7) |  |
|  | *Low anterior resection (n, %)* | 51 (33.6) | 42 (32.8) | 0.004 |
|  | *IPAA (n, %)* | 75 (49.3) | 41 (32.0) |  |
|  | *Sigmoid resection (n, %)* | 11 (7.2) | 13 (10.2) |  |
|  | *Diversion without resection (n, %)* | 8 (5.3) | 11 (8.6) |  |
|  | *Colonic resection (n, %)* | 7 (4.6) | 14 (10.9) |  |
|  | *Small bowel resection (n, %)* | 0 | 1 (0.6) |  |
|  | *Pull through with colo-anal anastomosis (n, %)* | 0 | 6 (4.7) |  |
| **Indication for**  **ileostomy** | *Diversion for primary disease or during primary surgery (n, %)* | 144 (94.7) | 106 (82.8) | 0.001 |
|  | *Secondary diversion for anastomotic leakage (n, %)* | 8 (5.3) | 22 (17.2) |  |
| **Time to stoma reversal** | *Mean weeks (± SD)* | 22.3 (±18.5) | 21.5 (±14.6) | 0.678 |
| **Colorectal surgeon performing or supervising surgery** | *Yes (n, %)*  *No (n, %)* | 81 (53.3)  71 (46.7) | 113 (88.3)  15 (11.7) | <0.001 |
| **Type of constructed** | *End-to-end anastomosis (n, %)* | 113 (74.3) | 36 (28.1) | <0.001 |
| **anastomosis** | *Side-to-side anastomosis (n, %)* | 24 (15.8) | 79 (61.7) |  |
|  | *Side-to-end or end-to-side anastomosis (n, %)* | 3 (2.0) | 6 (4.6) |  |
|  | *Unknown (n, %)* | 12 (7.9) | 7 (5.4) |  |
| **Anastomotic technique** | *Sewn (n, %)* | 131 (86.1) | 38 (29.6) | <0.001 |
|  | *Stapled (n, %)* | 16 (10.5) | 83 (64.8) |  |
|  | *Unknown (n, %)* | 5 (3.2) | 7 (5.4) |  |

BMI = Body Mass Index, ASA= American Society of Anaesthesiology, IPAA= ileal pouch-anal anastomosis.

| **Appendix 2** Postoperative morbidity after loop ileostomy closure | | ***Cohort A***  *June 2004- June 2010*  *(n=152)* | ***Cohort B***  *July 2010- Jan 2014*  *(n=128)* | *p-value* |
| --- | --- | --- | --- | --- |
| **Hospital stay** | *Median days (±IQR)* | 4.0 (3-7) | 5.0 (3-6) | 0.70 |
| **Wound infection** | *Number of patients (%)* | 4 (2.6) | 6 (4.7) | 0.52 |
| **Ileus** | *Number of patients (%)* | 12 (7.9) | 7 (5.5) | 0.48 |
| **Abscess** | *Intra-abdominal (n, %)* | 1 (0.7) | 3 (1.3) | 0.36 |
| **Anastomotic leakage** | *Number of patients (%)* | 8 (5.2) | 3 (2.3) | 0.24 |
| **Major morbidity** | *Clavien-Dindo ≥3 (n, %)* | 12 (7.9) | 5 (3.9) | 0.21 |
|  | *Clavien-Dindo grade 1-2 (n, %)* | 10 (6.6) | 9 (7.0) | 1.00 |
|  | *Clavien-Dindo grade 3 (n, %)* | 9 (5.9) | 1 (0.8) | 0.02 |
|  | *Clavien-Dindo grade 4 (n, %)* | 2 (1.3) | 4 (3.1) | 0.42 |
|  | *Clavien-Dindo grade 5 (n, %)* | 1 (0.7) | 1 (0.8) | 1.00 |

| **Appendix 3** Univariable analysis of risk factors for major morbidity after loop ileostomy closure | **Univariable analysis** | | |
| --- | --- | --- | --- |
|  | *OR* | *95% CI* | *p-value* |
| **Male gender** | 1.16 | 0.43-3.07 | 0.77 |
| **Age (years)** | 1.01 | 0.98-1.05 | 0.39 |
| **ASA Classification I**  **II**  **III** | -  0.49  1.64 | -  0.16-1.57  0.44-6.08 | -  0.23  0.46 |
| **Smoking** | 0.47 | 0.10-2.12 | 0.32 |
| **BMI** | 0.97 | 0.88-1.07 | 0.54 |
| **Malignant disease** (versus benign disease) | 0.56 | 0.19-1.61 | 0.28 |
| **Primary laparoscopic surgery** (versus open) | 0.51 | 0.18-1.48 | 0.22 |
| **Secondary diversion for anastomotic leakage**  (versus primary diversion) | 1.74 | 0.47-6.40 | 0.40 |
| **Colorectal surgeon** (versus any surgeon or resident) | 0.26 | 0.10-0.68 | 0.01 |
| **Stapled anastomosis** (versus hand-sewn) | 0.69 | 0.23-2.04 | 0.51 |
| **S-S anastomosis** (versus S-E, E-S, E-E anastomosis) | 1.58 | 0.57-4.34 | 0.38 |

BMI = Body Mass Index, ASA= American Society of Anaesthesiology, IPAA= ileal pouch-anal anastomosis.
